# The Prevalence of Uncontrolled Hypertension among Patients Taking Antihypertensive Medications and the Associated Risk Factors in North Palestine: A Cross-Sectional Study

**DOI:** 10.1155/2022/5319756

**Published:** 2022-08-25

**Authors:** Issa S. Alawneh, Ahmad Yasin, Samar Musmar

**Affiliations:** ^1^Department of Pediatrics, Najah University Hospital, Nablus, State of Palestine; ^2^Department of Pediatrics, Hamad Medical Cooperation, Doha, Qatar; ^3^Department of Family Medicine, Faculty of Medicine and Health Sciences, An-Najah National University, Nablus, State of Palestine

## Abstract

**Background:**

Uncontrolled hypertension (HTN) is a challenge for public health professionals all over the world. It is the leading and most important modifiable risk factor for coronary artery disease, congestive heart failure, stroke, renal diseases, and retinopathy. The aim of the present study was to estimate the prevalence of uncontrolled HTN among Palestinian hypertensive patients on treatment. In addition, the study aimed to explore the relationship between socio-demographic and clinical factors with HTN control as well as establish a comprehensive literature review for similar studies.

**Methods:**

A cross-sectional study was conducted. 218 hypertensive patients who met the inclusion criteria were included in the study.

**Results:**

HTN is not adequately controlled in over 60% of treated patients. Factors that were linked to uncontrolled HTN and were statistically significant as per this study were diabetes (*p*=0.010), high BMI (*p*=0.009), smoking (*p* < 0.0001), lower educational level (*p*=0.002), and monotherapy (*p*=0.004).

**Conclusion:**

The results suggest that effective efforts on improving HTN control are strongly needed. The efforts need to target hypertensive patients who are also smokers, diabetics, having a low education level, and have a higher-than-normal BMI.

## 1. Introduction

Hypertension (HTN) is a major medical problem which affects over one billion individuals and leads to ∼1.7 million deaths per year worldwide [1–3]. Uncontrolled HTN presents a challenge for the primary health care (PHC) setting. The reasons for this global epidemic of uncontrolled HTN are not well understood, but barriers to the adequate control of HTN are likely to be complex and arise from a combination of factors related to patients, physicians, and health systems [4, 5].

Subjects with HTN are twice as likely to develop coronary artery disease and are four and seven times more likely to experience congestive heart failure, cerebrovascular disease, and stroke, respectively, compared to normotensive subjects [6]. In addition, long-standing HTN may cause proteinuria and progressive renal failure [7]. Given the rising prevalence of HTN in developing countries, the treatment and control of high blood pressure (BP) are critical for reducing cardiovascular disease risk and diminishing the associated burden of illness.

Evidence has shown that age, gender, nonadherence to a low-salt diet, obesity, smoking, and the number of medications taken were among the major contributing factors to uncontrolled HTN [8–10]. In the Palestinian population, the prevalence of HTN is 27.6%, affecting a higher proportion of men than women [11]. However, there is a lack of research focusing specifically on patients with uncontrolled HTN to explore the associated risk factors and improve HTN control in the Palestinian population. In this study, we set out to estimate the prevalence of uncontrolled HTN among patients taking anti-HTN medications and the associated risk factors in northern Palestine.

## 2. Methodology

### 2.1. Study Design and Setting

A cross-sectional study was conducted at the Al-Makhfeya Palestinian Ministry of Health (MOH) PHC center in Nablus, a large city located in the north of Palestine. This PHC center was chosen because it receives patients from all over northern Palestine who are either physician-referred or self-referred.

### 2.2. Study Population

The target population comprised HTN patients taking anti-HTN medications who agreed to participate in the study and who met the following selection criteria: aged ≥18 years, taking at least one pharmacological anti-HTN medication for at least 3 months. We excluded patients who were not aware of having HTN or who were not taking pharmacological anti-HTN medication.

### 2.3. Study Samples

We used the convenience sampling technique. Prior to recruitment, we engaged patients with HTN who came to the internal medicine and general practice clinics for follow-up or any other medical reason randomly and invited them to participate in our study.

### 2.4. Data Collection Procedure

We initially obtained study approval from the An-Najah National University Institution Review Board (IRB) and the Palestinian MOH. Once permissions were granted, daily visits to internal medicine and general practice clinics at the Al-Makhfeya PHC center were initiated. Data collection took place between January 24 and February 28, 2013. The two principal investigators served as interviewers; they started by thoroughly explaining the research and its purpose to each participant before taking verbal consent. Next, a checklist questionnaire was completed, including socio-demographic and clinical factors associated with HTN. Subsequently, anthropometric measurements (weight, height, and body mass index (BMI)) were obtained, and a BP reading was recorded.

### 2.5. Study Variables

The dependent variable was uncontrolled HTN. The independent variables were socio-demographic characteristics (i.e., age, gender, marital status, and education level), associated risk factors for uncontrolled HTN (i.e., low salt diet adherence and smoking status), and clinical characteristics (i.e., BMI and the number of anti-HTN drugs taken).

### 2.6. Study Term Definitions

Adherence to a low-salt diet: the participant's habits of avoiding or decreasing the addition of salt to cooked meals and avoiding foods with a high salt content in general.

Diabetic mellitus: self-reported diabetes, the use of hypoglycemic agents, or both.

HTN has a documented diagnosis of HTN (i.e., BP ≥140/90 mmHg).

Uncontrolled HTN. a BP ≥140/90 mmHg (determined using a digital sphygmomanometer) for adults with HTN but without diabetes mellitus or chronic kidney disease, for at least three consecutive follow-up measurements; and a BP ≥130/80 mmHg (determined using digital sphygmomanometer) for adults with HTN, diabetes mellitus, and chronic kidney disease, for at least three consecutive follow-up measurements.

### 2.7. Checklist Questionnaire

This was divided into five parts:

First part: checklist for the inclusion and exclusion criteria
Second part: socio-demographic factors for participants (i.e., age, gender, marital status, and education level)
Third part: assessment of associated risk factors, including diabetes mellitus and smoking
Fourth part: assessment of BP control
  - Questions relating to BP management with anti-HTN medication (i.e., the number of anti-HTN medications currently taken)
  - Questions relating to salt restriction habits
Fifth part: completed using anthropometric and BP measurements

### 2.8. Anthropometric Measurements

  Weight: each participant's weight (to the nearest 0.5 kg) was recorded using an ordinary nonelectronic portable weighing scale, which was standardized and well calibrated. Weight was measured while the participant was wearing light clothing and no shoes.
  Height: height measurements (to the nearest cm) were performed on an uncarpeted area using an ordinary measuring tape. Subjects were requested to stand upright without shoes and with their back against the wall, heels together, and eyes directed forward.
  BMI: BMI was calculated as weight in kg divided by the height in meters squared (kg/m^2^). BMI values were categorized as follows: normal (BMI values <25), overweight (BMI values 25–30), and obese (BMI values >30), according to the previously published criteria [12].

### 2.9. BP Measurement

BP measurement was performed by two trained 6^th^ year medical student researchers. BP was measured in the right upper arm, while the participant was in a seated position. A mercury sphygmomanometer that was standardized and well-calibrated (with a 14 cm cuff) was used to measure BP after the participants had rested for 5 minutes. An average of three BP readings was recorded to the nearest 2 mmHg. Systolic BP was recorded at the appearance of the relevant sounds (first Korotkoff sounds), and diastolic BP was recorded at the disappearance of the relevant sounds (fifth Korotkoff sounds) [13].

### 2.10. Reliability and Validity of the Research Tools

All the studied variables and questions were selected according to the study objectives and a literature review. The questionnaire was pretested in a pilot study, and all necessary changes were made prior to study initiation. The questionnaire was reviewed by the study supervisor.

### 2.11. Potential Confounders

The participants' medical history was collected, and the examination was performed by the same two researchers to reduce bias. All anthropometric and BP readings were measured using the same tools.

### 2.12. Statistical Analysis

Statistical analysis was conducted using the Statistical Package for Social Sciences (SPSS) version 17. Since all the independent variables were categorical, the Chi-square test was used to perform a univariate analysis to study the relationship between each independent variable and the BP control. When the *P* value was less than 0.05, the relationship was considered statistically significant. Multivariate logistic regression was used to re-examine the significance of the relationship between BP control and independent variables, which were found to be statistically significant by univariate analysis.

### 2.13. Ethical Considerations

The An-Najah National University IRB approved the study proposal on January 23, 2013. All steps in the study were performed after the IRB approval was granted and in accordance with the Declaration of Helsinki and its amendments. Written consent was also obtained from the Palestinian MOH. Verbal consent was obtained from all study subjects. The data were treated in a private and confidential manner during collection, entry, and analysis.

### 2.14. Data Availability

The datasets generated and analysed during the present study are not publicly available due to a data management agreement with the An-Najah National University; however, these data can be obtained from the corresponding author on reasonable request.

## 3. Results

Two hundred and eighteen patients with HTN, who met the inclusion criteria and were under follow-up at the internal medicine and general practice clinics in the al-Makhfeya PHC center (Nablus, Palestine), were enrolled in this study.

### 3.1. Socio-Demographic Characteristics

60% of the study subjects were women under the age of 60 years old with only a basic level of education. Almost 70% of the participants were married (Table 1).

### 3.2. Behavioral and Clinical Risk Factors for Uncontrolled HTN

**Figure 1:**
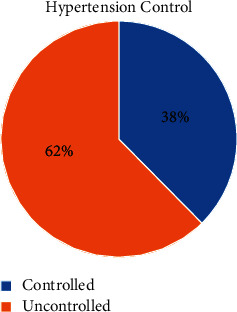
Prevalence of uncontrolled HTN.

Of the 218 participants, 85% had a higher-than-normal BMI and half had diabetes mellitus. In addition, almost half of the patients with HTN consumed dietary salt without restriction, about one-third were smokers, and a half used only one anti-HTN medication (Table 2). When the criteria for defining uncontrolled HTN were applied, 136 of the study samples were found to have uncontrolled BP, meaning that the prevalence of uncontrolled HTN was 62.4% (Figure 1).

### 3.3. Uncontrolled HTN and Associated Factors

HTN control was studied in relation to many socio-demographic variables, including age, gender, marital status, and education level, as shown in Table 3.

According to the results in Table 3, there was a statistically significant relationship between uncontrolled HTN and the education level of participants (*P*=0.021). However, there was no significant relationship between BP control and other studied socio-demographic factors, including age, gender, or marital status. BP control was also studied in relation to behavioral and medical variables, including diabetes mellitus, salt restriction habits, BMI, smoking, and the number of anti-HTN drugs taken, as shown in Table 4.

Table 4 shows that there was a statistically significant relationship between BP control and the following variables: diabetic status (*P*=0.025), BMI (*P*=0.027), smoking (*P*=0.010), and the number of anti-HTN medications used (*P*=0.037). However, salt restriction did not have a significant effect on BP control. In other words, uncontrolled HTN was higher in participants who were diabetic, had higher-than-normal BMI, were smokers, and had used monotherapy to treat their HTN.

To adjust for participants' confounding factors, we re-examined factors that were found to have a statistically significant relationship by univariate analysis using multivariate logistic regression as shown in Table 5.

Table 5 shows that HTN patients with diabetes mellitus were twice as likely to exhibit poor BP control, compared to nondiabetic individuals (OR = 1.905, CI = 1.093–3.318). Having a higher-than-normal BMI was also significant and positively correlated with uncontrolled BP (OR = 2.451, CI = 1.145–5.247). Smoking (OR = 4.068, CI = 1.214–4.445), a lower level of education (OR = 1.868, CI = 1.062–3.286), and receiving anti-HTN monotherapy (OR = 1.860, CI = 1.038–3.143) had a significant negative impact on BP control.

## 4. Discussion

The inadequate control of high BP presents a significant challenge for the health system in Palestine. In our cross-sectional study, the majority of HTN patients could not achieve their target BP. It is likely that this inability to control BP has a significant impact on the mortality and morbidity associated with coronary artery disease, stroke, renal disorders, and other HTN-related diseases [14]. Uncontrolled HTN not only has considerable consequences in terms of patient mortality and morbidity but also in terms of health care costs [15]. The high prevalence of inadequately managed HTN suggests that a significant number of cardiovascular events could be prevented by improving BP control.

The Third National Health and Nutrition Examination Survey (NHANES III) estimated that the control of HTN could prevent the onset of coronary heart disease in 19%–56% and 31%–57% of men and women, respectively [16]. The prevalence of uncontrolled HTN in our Palestinian study was 62.4%; the situation in other Arab countries is not any better. Reports from Saudi Arabia and Bahrain showed an HTN control rate of 25% and 16.5%, respectively [17, 18]. BP control rates vary from one country to another, ranging from 5.4% in Korea to 58% in Barbados, with a worldwide average of around 30%. These figures demonstrate the difficulty of achieving satisfactory BP control worldwide [19].

In our study, approximately 31% of patients who were only taking a single anti-HTN medication could achieve the target BP. Our findings were compatible with research originating from the United States (US), which demonstrated that BP targets were reached in only 30%–40% of patients taking a single anti-HTN drug [20]. However, we found that patients who were receiving combination anti-HTN pharmacological therapy were better at controlling their HTN than those on monotherapy (*P*=0.004). This finding is in agreement with the HTN management and control guidelines (JNC-7) [1].

Patients who have both HTN and diabetes mellitus are at a higher risk of experiencing cardiovascular events compared to nondiabetic HTN patients [21]. We found that diabetic patients with HTN demonstrated remarkably poor BP control (*P*=0.010). Research from Saudi Arabia has shown similar findings [22]. Such results could be related to the strict definition of controlled BP in diabetic patients (<130/80 mmHg). It is likely that this BP target may not yet be fully employed in routine practice.

Another potentially important observation in this study was the relationship between poor BP control and having a higher-than-normal BMI (*P*=0.009), which was consistent with findings from a cross-sectional study carried out in Sweden [23]. These findings highlight the need for health care providers in PHC settings to be aware of the importance of obesity prevention and treatment as part of HTN management in order to achieve better BP control.

Although our study failed to show any statistically significant association between the control of HTN and age, many studies from the US and Spain found that old age was a significant risk factor for uncontrolled HTN [23, 24]. This may be attributed to the conservative attitude of physicians in prescribing more drugs to attain the required BP values at this age. Likewise, gender was not a significant factor affecting BP control in our study, which was consistent with an Italian study [25]. Although we could not find a statistically significant relationship between gender and BP control, the prevalence of HTN control was higher among women than men; similar conclusions were reached by researchers in France, Finland, and South Korea [26–28]. We also found that marital status was not a significant factor affecting BP control. In contrast, smoking was a highly significant factor in BP control (*P* < 0.0001), which was in accordance with the work of Jo I et al. [29]. Therefore, physicians may need to be especially vigilant about BP control in patients who smoke.

In the present study, we evaluated the pattern of dietary salt intake and found that the highest proportion of patients with uncontrolled HTN adhered to salt restriction, although this difference was not significant. On the contrary, a study from the US found that salt intake played an important role in the development and control of HTN [30]. On studying the relationship between HTN control and educational level, we observed the highest proportion of uncontrolled HTN was among secondary school and college graduates, which was statistically significant (*P*=0.002). The same result was reported in an Iraqi study [25]. We can surmise that the poor medical knowledge of patients with a lower education level could limit their awareness on the importance of strict BP control.

## 5. Conclusions

The present study concluded that HTN is not adequately controlled in over 60% of treated patients. This was explained by different factors, including comorbidities like diabetes mellitus and obesity, as well as inadequate pharmacological treatment regimens. Moreover, smoking and lower education level were significant contributors. Indeed, improving the quality of HTN care is a public health priority, and effective efforts need to be made. Future plans should be implemented in the Palestinian PHC system focusing on HTN patients with diabetes mellitus who smoke, with a particular focus on overweight individuals. Furthermore, patients with low education levels should be better informed about the importance of managing their BP and HTN. We recommend that further studies be performed on a bigger scale, using a larger sample size and including PHC centers from other cities to give a more representative view of the Palestinian population.

### 5.1. Study Strengths

To our knowledge, this study is the first of its kind to measure the prevalence of uncontrolled BP in the Palestinian population. Our work highlights a crucially important public health issue. We used internationally accepted criteria to generate valuable results on the prevalence and risk factors associated with uncontrolled HTN that further studies could rely on.

### 5.2. Study Limitations

This was a cross-sectional study, so any interpretation should be avoided regarding causal relationships between the previously mentioned factors.

This study was conducted on patients attending a PHC center in Nablus, who may have specific socio-economic characteristics that may not be representative of the whole Palestinian population. It might therefore have been better to conduct the study on a larger, more diverse, and representative group of participants.

## Figures and Tables

**Table 1:** Socio-demographic characteristics of the study sample.

Variables	Category	Frequency	Percentage
Age (years)	Less than 60	135	61.9
	60 and more	83	38.1

Gender	Male	87	39.9
	Female	131	60.1

Education	Primary school and less	137	62.8
	Secondary school and college	81	37.2

Marital status	Married	159	72.9
	^*∗*^Nonmarried	59	27.1

	Total	218	100

^*∗*^Nonmarried (single, divorced, or widowed); *n* = 218.

**Table 2:** Medical and health characteristics of the study sample.

Variables	Category	Frequency	Percentage
Smoking	Smoker	65	29.8
	Nonsmoker	153	70.2

Diabetes	Diabetic	120	55
	Nondiabetic	98	45

Salt restriction habit	No salt restriction	93	42.7
	Salt restriction	125	57.3

BMI (kg/m^2^)	≥30	79	36.2
	25–30	107	49.1
	≤25	32	14.7

Number of antihypertensive drugs	Mono therapy	113	51.8
	Combination therapy	105	48.2
	Total	218	100

*n* = 218.

**Table 3:** Relationship of socio-demographic variables with HTN control applying the Chi-square test.

Variables	Category	Hypertension		X^2^	*P* value^*∗*^
		Uncontrolled	Controlled		
Age (years)	Less than 60	85 (63%)	50 (37%)	0.050	0.886
	60 and more	51 (61.4%)	32 (38.6%)		

Gender	Male	58 (66.7%)	29 (33.3%)	1.131	0.180
	Female	78 (59.5%)	53 (40.5%)		

Education	Primary school and less	93 (67.9%)	44 (32.1%)	4.749	0.021
	Secondary school and college	43 (53.1%)	38 (46.9%)		

Marital status	Married	56 (70.9%)	23 (29.1%)	1.741	0.210
	^*∗∗*^Nonmarried	66 (61.9%)	41 (38.3%)		

^*∗*^ *P* value of 0.05 or less was considered statistically significant. ^*∗∗*^Nonmarried (single, divorced, or widowed).

**Table 4:** Relationship of medical and health variables with HTN control applying the Chi-square test.

Variables	Category	Hypertension		X^2^	*P* value^*∗*^
		Uncontrolled	Controlled		
Diabetes	Diabetic	83 (69.2%)	37 (30.8%)	5.231	0.025
	Nondiabetic	53 (54.1%)	45 (45.9%)		

Salt restriction habit	No salt restriction	62 (66.7%)	31 (33.3%)	1.267	0.163
	Salt restriction	74 (59.2%)	51 (40.8%)		

BMI (kg/m^2^)	≥30	56 (70.9%)	23 (29.1%)	7.191	0.027
	25–30	66 (61.7%)	41 (38.3%)		
	≤25	14 (43.8%)	18 (56.2%)		

Smoking	Smoker	49 (75.4%)	16 (24.6%)	1.741	0.210
	Nonsmoker	87 (56.9%)	66 (43.1%)		

Number of antihypertensive drugs	Mono therapy	78 (69.1%)	35 (30.9)	4.410	0.037
	Combination therapy	58 (55.3%)	47 (44.7%)		

^*∗*^ *p* value of 0.05 or less was considered statistically significant.

**Table 5:** Relationship between HTN control and selected risk factors after adjustment for confounding factors.

Variables	Category	Hypertension		Odds ratio	CI	P value^*∗∗*^
		Uncontrolled	Controlled			
Diabetes	Diabetic	83 (69.2%)	37 (30.8%)	1.905	1.093–3.318	0.010
	Nondiabetic	53 (54.1%)	45 (45.9%)			

BMI (kg/m^2^)	≥30	56 (70.9%)	23 (29.1%)	2.451	1.145–5.247	0.009
	25–30	66 (61.7%)	41 (38.3%)			
	≤25	14 (43.8%)	18 (56.2%)			

Smoking	Smoker	49 (75.4%)	16 (24.6%)	4.068	1.214–4.445	<0.0001
	Nonsmoker	87 (56.9%)	66 (43.1%)			

Number of antihypertensive drugs	Mono therapy	78 (69.1%)	35 (30.9)	1.860	1.038–3.143	0.004
	Combination therapy	58 (55.3%)	47 (44.7%)			

CI: confidence interval = 95%. ^*∗∗*^*P* value of 0.05 or less was considered statistically significant.

## Data Availability

The datasets generated and analysed during the present study are not publicly available due to participants' private policies and research regulation agreement related to Al-Najah National University but are available from the corresponding author on reasonable request.
